# Supplementary material for: Forbidden links, trait matching and modularity in plant-hummingbird networks: Are specialized modules characterized by higher phenotypic floral integration?
Source: PeerJ. 2021 Mar 10;9:e10974. doi: 10.7717/peerj.10974 (PMC7955668; doi:10.7717/peerj.10974)
Supplement: Supplemental Information 5 — Each plant species is organized according to the module to which it belongs: Chlorestes candida (Ch. candida) and Amazilia tzacatl (A. tzacatl) module, Phaethornis longirostris (P. longirostris) module and Phaethornis striigularis (P. striigularis) module. The phenotypic integration index (PINT), the percentage of maximum possible integration (RelPINT) and the number of observations used (N) is reported for each plant species and module average. [file peerj-09-10974-s005.docx]

| **Module** | **Plant species** | **PINT** | **RelPINT** | **N** |
| --- | --- | --- | --- | --- |
| *Amazilia tzacatl, Chlorestes candida* | *Androlepis skinneri* | 0.23 | 7.52 | 29 |
| *Amazilia tzacatl, Chlorestes candida* | *Catopsis berteroniana* | 1.17 | 29.22 | 12 |
| *Amazilia tzacatl, Chlorestes candida* | *Heliconia latispatha* | 0.64 | 15.89 | 23 |
| *Amazilia tzacatl, Chlorestes candida* | *Heliconia librata* | 0.74 | 18.41 | 21 |
| *Amazilia tzacatl, Chlorestes candida* | *Psychotria poeppigiana* | 0.92 | 30.76 | 13 |
| **Module average** |  | **0.74** | **20.36** | **98** |
| *Phaethornis longirostris* | *Billbergia viridiflora* | 0.90 | 22.48 | 5 |
| *Phaethornis longirostris* | *Bromelia pinguin* |  |  |  |
| *Phaethornis longirostris* | *Costus pictus* | 1.23 | 30.71 | 6 |
| *Phaethornis longirostris* | *Costus scaber* | 1.23 | 30.72 | 11 |
| *Phaethornis longirostris* | *Erythrina folkersii* | 0.71 | 17.63 | 11 |
| *Phaethornis longirostris* | *Heliconia aurantiaca* | 0.77 | 19.12 | 14 |
| *Phaethornis longirostris* | *Heliconia collinsiana* | 0.09 | 2.21 | 178 |
| *Phaethornis longirostris* | *Heliconia wagneriana* | 1.03 | 25.64 | 48 |
| *Phaethornis longirostris* | *Justicia aurea* | 1.87 | 46.75 | 40 |
| *Phaethornis longirostris* | *Malvaviscus arboreus* | 0.39 | 9.81 | 39 |
| *Phaethornis longirostris* | *Vriesea heliconioides* |  |  |  |
| **Module average** |  | **0.91** | **22.79** | **39** |
| *Phaethornis striigularis* | *Aechmea tillandsioides* |  |  |  |
| *Phaethornis striigularis* | *Aechmea bracteata* | 1.06 | 26.15 | 13 |
| *Phaethornis striigularis* | *Calathea lutea* | 0.27 | 6.78 | 22 |
| *Phaethornis striigularis* | *Odontonema callistachyum* | 0.59 | 14.79 | 12 |
| *Phaethornis striigularis* | *Odontonema tubaeforme* | 0.32 | 7.92 | 25 |
| *Phaethornis striigularis* | *Palicourea triphylla* | 1.45 | 36.28 | 8 |
| *Phaethornis striigularis* | *Stromanthe macrochlamys* | 1.26 | 31.46 | 25 |
| *Phaethornis striigularis* | *Tillandsia bulbosa* | 0.88 | 21.88 | 10 |
| *Phaethornis striigularis* | *Tillandsia pruinosa* |  |  |  |
| *Phaethornis striigularis* | *Tillandsia streptophylla* | 0.75 | 18.73 | 9 |
| **Module average** |  | **0.82** | **20.50** | **16** |
