# Supplementary material for: Forbidden links, trait matching and modularity in plant-hummingbird networks: Are specialized modules characterized by higher phenotypic floral integration?
Source: PeerJ. 2021 Mar 10;9:e10974. doi: 10.7717/peerj.10974 (PMC7955668; doi:10.7717/peerj.10974)
Supplement: Supplemental Information 6 — For each plant species of both habitats, floral measures, nectar metrics, family identity and legitimate hummingbird visitors are shown. [file peerj-09-10974-s006.docx]

| **Species** | **Family** | **Habitat** | **Corolla length (mm)** | **Curvature (degrees)** | **Nectar volume (µl)** | | **Nectar concentration (°Bx)** | **Legitimate visitors** | |
| --- | --- | --- | --- | --- | --- | --- | --- | --- | --- |
| *Justicia aurea* | Acanthaceae | Rainforest | 66.81±3.86 (N=97) | 8.75±1.08 (N=5) | 10.93±5.87 (N=46) | | 20.26±1.78 (N=45) | *P. longirostris* | |
| *Odontonema callistachyum* | Acanthaceae | Rainforest | 16.29±0.74 (N=30) | 8.86±1.51 (N=4) | 1.54±2.07 (N=30) | | 17.21±1.18 (N=12) | *P. striigularis* | |
| *Odontonema tubaeforme* | Acanthaceae | Rainforest | 21.75±1.89 (N=42) | 20.81±1.77 (N=5) | 3.79±2.25 (N=25) | | 19.78±1.70 (N=25) | *P. striigularis* | |
| *Aechmea bracteata* | Bromeliaceae | Savanna | 10.10±0.61 (N=25) | 0 (N=5) | 2.54±1.78 (N=13) | | 20.88±2.10 (N=13) | *Ch. candida, A. tzacatl, P. striigularis* | |
| *Aechmea tillandsioides* | Bromeliaceae | Rainforest | 22.79±2.08 (N=11) | 0 (N=3) | 11.46±3.31 (N=11) | | 23.86±1.27 (N=11) | *A. tzacatl, P. longirostris* | |
| *Androlepis skinneri* | Bromeliaceae | Savanna | 10.62±0.74 (N=86) | 0 (N=4) | 21.60±1.02 (N=29) | *A. candida, A. tzacatl, P. longirostris, P. striigularis* | | |  |
| *Billbergia viridiflora* | Bromeliaceae | Rainforest | 42.52±0.69 (N=15) | 18.85±1.35 (N=2) | 20.46±6.16 (N=5) | | 27.5±1.46 (N=5) | *P. longirostris* | |
| *Bromelia pinguin* | Bromeliaceae | Rainforest | 31.54±1.33 (N=3) |  |  | |  | *P. longirostris* | |
| *Catopsis berteroniana* | Bromeliaceae | Savanna | 12.8±1.97 (N=24) | 0 (N=6) | 19.18±8.8.16 (N=13) | | 18.08±1.20 (N=12) | *Ch. candida, A. tzacatl, P. longirostris, P. striigularis* | |
| *Tillandsia bulbosa* | Bromeliaceae | Savanna | 36.04±8.90 (N=10) | 9.12 (N=1) | 4.41 (N=1) | | 22.5 (N=1) | *P. striigularis* | |
| *Tillandsia pruinosa* | Bromeliaceae | Savanna | 19.98±1.99 (N=4) | 8.2 (N=1) |  | |  | *P. striigularis* | |
| *Tillandsia streptophylla* | Bromeliaceae | Savanna | 39.59±4.42 (N=14) | 8.17 (N=1) | 5.86±3.21 (N=9) | | 22.83±2.08 (N=9) | *P. striigularis* | |
| *Vriesea heliconioides* | Bromeliaceae | Rainforest | 33.01±0.02 (N=2) | 9.87 (N=1) | 4.42±2.21 (N=2) | | 23.5±3.53 (N=2) | *P. longirostris* | |
| *Costus pictus* | Costaceae | Rainforest | 78.25±2.06 (N=3) | 12.1 (N=1) | 44.23±22.17 (N=7) | | 33.64±3.27 (N=7) | *P. longirostris* | |
| *Costus scaber* | Costaceae | Rainforest | 51.60±15.85 (N=47) | 21.45±1.08 (N=6) | 33.05±22.34 (N=16) | | 25.53±5.74 (N=16) | *P. longirostris* | |
| *Erythrina folkersii* | Fabaceae | Rainforest | 76.03±2.74 (N=21) | 0 (N=5) | 16.52±7.36 (N=11) | | 17±2.83 (N=12) | *P. longirostris* | |
| *Heliconia aurantiaca* | Heliconiaceae | Rainforest | 51.87±2.05 (N=22) | 18.81±0.90 (N=2) | 40.14±13.93 (N=14) | | 22.36±3.36 (N=14) | *P. longirostris* | |
| *Heliconia collinsiana* | Heliconiaceae | Rainforest | 52.8±1.20 (N=180) | 18.49±2.37 (N=16) | 58.39±31.67 (N=178) | | 27.88±4.85 (N=68) | *P. longirostris* | |
| *Heliconia latispatha* | Heliconiaceae | Rainforest | 36.54±0.92 (N=34) | 5.55±0.08 (N=3) | 75.85±27.73 (N=23) | | 24.76±1.73 (N=23) | *Ch. candida, A. tzacatl, P. longirostris, P. striigularis* | |
| *Heliconia librata* | Heliconiaceae | Rainforest | 19.69±1.83 (N=79) | 5.8±0.43 (N=5) | 21.62±9 (N=21) | | 24.33±2.13 (N=21) | *Ch. candida, A. tzacatl, P. longirostris, P. striigularis* | |
| *Heliconia wagneriana* | Heliconiaceae | Rainforest | 62.13±2.22 (N=101) | 12.73±0.39 (N=2) | 21.01±13.27 (N=52) | | 24.06±4.81 (N=49) | *P. longirostris* | |
| *Malvaviscus arboreus* | Malvaceae | Rainforest | 41.42±2.51 (N=42) | 0 (N=7) | 18.33±12.37 (N=44) | | 20.21±4.13 (N=44) | *P. longirostris* | |
| *Calathea lutea* | Marantaceae | Rainforest | 29.87±2.78 (N=29) | 12.88±0.29 (N=11) | 14.1±3.51 (N=22) | | 36.82±1.18 (N=22) | *P. striigularis* | |
| *Stromanthe macrochlamys* | Marantaceae | Rainforest | 7.61±0.68 (N=65) | 0 (N=8) | 3.75±1.27 (N=25) | | 24.38±2.38 (N=25) | *P. striigularis* | |
| *Palicourea triphylla* | Rubiaceae | Savanna | 11.17±1.94 (N=38) | 5.84±0.41 (N=4) | 8.45±2.46 (N=8) | | 21.25±1 (N=8) | *P. striigularis* | |
| *Psychotria poeppiginiana* | Rubiaceae | Savanna | 15.57±2.77 (N=100) | 0 (N=6) | 7.32±4.89 (N=14) | | 24.32±3.29 (N=14) | *Ch. candida, A. tzacatl, P. striigularis* | |
